# fluEvidenceSynthesis: An R package for evidence synthesis based analysis of epidemiological outbreaks

**DOI:** 10.1371/journal.pcbi.1005838

**Published:** 2017-11-20

**Authors:** Edwin van Leeuwen, Petra Klepac, Dominic Thorrington, Richard Pebody, Marc Baguelin

**Affiliations:** 1 Respiratory Diseases Department, Public Health England, London, United Kingdom; 2 National Heart and Lung Institute, Imperial College London, London, United Kingdom; 3 School of Public Health, Imperial College London, London, United Kingdom; 4 London School of Hygiene and Tropical Medicine, London, United Kingdom; Hebrew University of Jerusalem, ISRAEL

## Abstract

Public health related decisions often have to balance the cost of intervention strategies with the benefit of the reduction in disease burden. While the cost can often be inferred, forward modelling of the effect of different intervention options is complicated and disease specific. Here we introduce a package that is aimed to simplify this process. The package allows one to infer parameters using a Bayesian approach, perform forward modelling of the likely results of the proposed intervention and finally perform cost effectiveness analysis of the results. The package is based on a method previously used in the United Kingdom to inform vaccination strategies for influenza, with extensions to make it easily adaptable to other diseases and data sources.

This is a *PLOS Computational Biology* Software paper.

## Introduction

In-depth cost effectiveness analysis of disease intervention strategies has been difficult to perform due to a variety of reasons, including the inherent complexity of disease dynamics, lack of data and difficulty in predicting the success of the proposed interventions. Currently available tools for analysing epidemiological data mainly focus on statistical analyses and simple regression models [1–3], which is not sufficient to model the population wide effects of proposed interventions, which include both direct and indirect effects. The exception here is amei, an R package for optimising vaccination strategies in using an adaptive management framework while evaluating costs of an underlying stochastic epidemiological model [4], but this package mainly relies on the use of a single (high quality) source of information. Synthesising information from different sources of data and performing a detailed cost effectiveness analysis of control strategies is not supported by the currently available packages.

In an effort to fill this gap and make sophisticated cost effectiveness analysis methods more accessible and standardised we have developed an R package that simplifies the process. Focusing on influenza, we present a package that offers detailed cost effectiveness analyses of intervention strategies in a Bayesian inference framework that applies evidence synthesis techniques to combine the available evidence from multiple data sources that can be stratified by age and risk status and combined with age-stratified mixing patterns. The implementation is based on a Susceptible Exposed Infectious Removed (SEIR) model previously developed for pandemic influenza [5] and later used for assessing the cost/benefit of changes in the seasonal influenza vaccine programme in the United Kingdom, and adapted to make it more generally applicable to other settings [6, 7].

The package is particularly suitable for modelling and evaluating intervention strategies for directly transmissible diseases that use proxy data for incidence, such as influenza-like-illness (ILI) counts for influenza and respiratory syncytial virus (RSV). The package deals with the whole pipeline, starting from re-organising users’ different data sources to standardise their format to be compatible with the package, to modelling, Bayesian inference, analysing vaccination scenarios and cost effectiveness. Crucially, it includes a number of high level functions that can do a particular analysis, and also provides access to the low level functions that are needed if the user wants to adapt the analysis and tailor it to their specific needs (e.g. user-provided data may be different than the data we have accessible).

## Design and implementation

**Fig 1:**
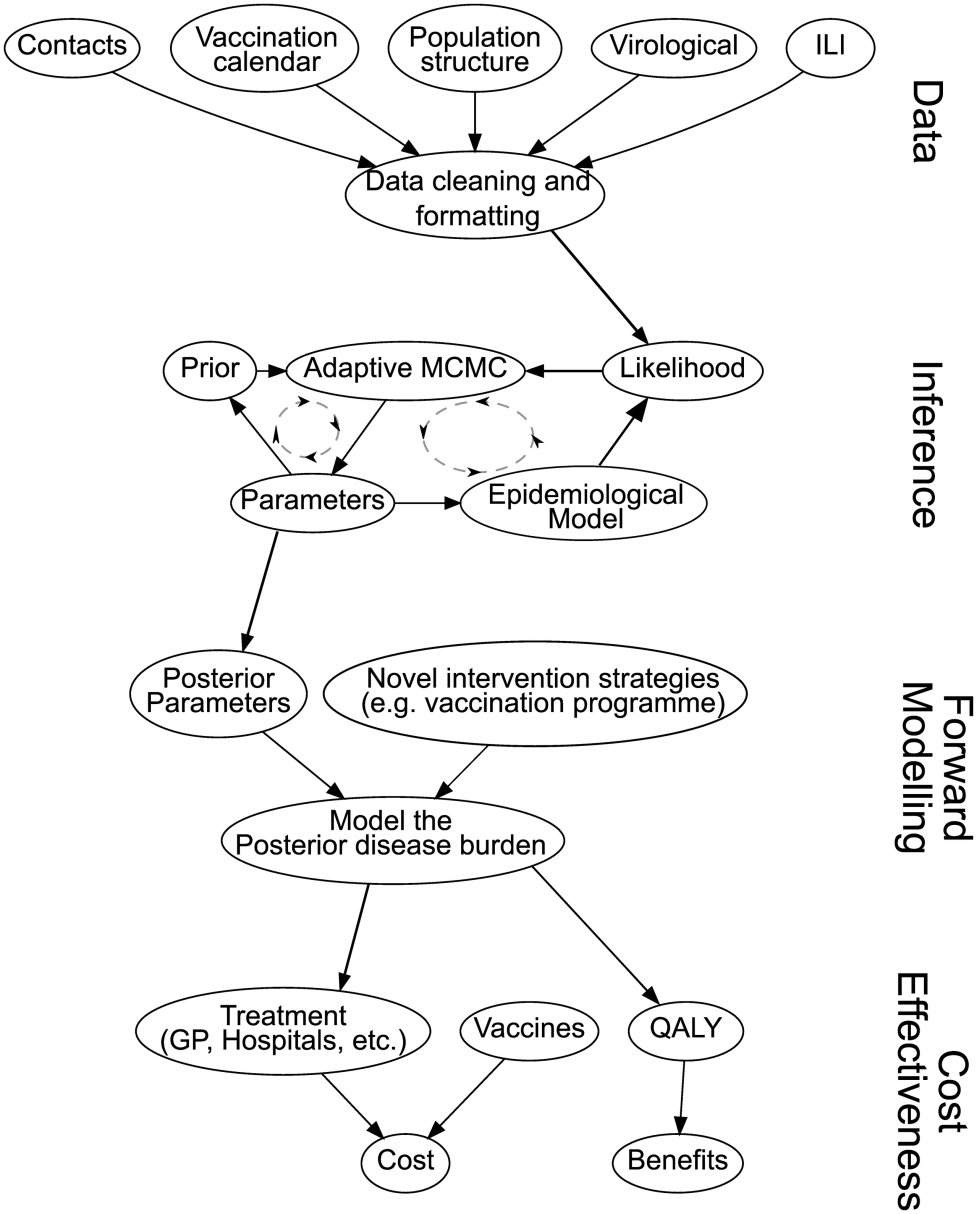
Workflow for the fluEvidenceSynthesis package.

Fig 1 illustrates the general workflow of the package. The package takes data on the influenza outbreak and related data, such as contact rates and demographic structure, as its input, which can then be organised into different age and risk groups. Given a set of parameters and considering the underlying model, we calculate the likelihood of observing these data. Given the data and the likelihood function, the package uses an adaptive MCMC algorithm to derive the posterior distribution of parameter values of the underlying epidemiological model. These posterior parameter values can be used to explore alternative intervention strategies, and for the cost-benefit analysis by using the functionalities of the forward modelling.

### Data representation

Data analysed in the package need to be transformed into a standardised format. The default input is in form of weekly data with each row representing the data for that week. Other dates can be used when using the lower level functions provided by the package. Columns contain the data stratified by age group. If the data are also stratified by risk group then the first group of columns will hold the data for one risk group, stratified by age group. The next set of columns will be the next risk group by age group, etc. The input as used in the examples consist of (see Fig 1):

- Weekly ILI counts, stratified by age in separate columns.
- Weekly virological data, stratified by age in separate columns.
- Weekly vaccination data, stratified by risk group and by age as explained above.
- Population size by age.
- Contact data by age group. The example data included in the package are based on the POLYMOD study [8].

### Functionalities

#### Stratifying the data

General functions are provided as part of the package to stratify the data and convert them to a suitable formatting for model fitting (e.g stratify_by_age(…)) and easily stratify the data into different user-specified age and risk groups. The choice of age groups and risk groups is flexible and depends on the data provided and the goal of the user. For example, for the UK the influenza occurrence data are separated into 5 age groups, while the vaccination data are divided into 7 different age groups, so it was decided to model 7 different age groups, but transform them into 5 before performing the fitting [6].

#### Vaccination scenarios

The package defines vaccination scenarios as a combination of the effectiveness of the vaccine against the dominant strain and the coverage over time. The effectiveness of the influenza vaccine is highly dependent on the match between the vaccine strain and the dominant strain circulating in the population. Influenza vaccine effectiveness is also age dependent, with the vaccine generally being less effective for the older age groups (e.g. because of immunosenescence). Vaccine coverage is determined by the vaccination programme. In most countries influenza vaccination is aimed at individuals aged 65 years and above, and at the high risk groups. In the United States and, more recently, in the UK the seasonal influenza vaccination programme was extended to include healthy children [7, 9]. The period during which the vaccination programme is carried out is also country-dependent. In the UK vaccination starts in October and runs until the end of January, with the largest uptake rate during the first month. The function as.vaccination.calendar(…) takes the effectiveness and coverage by age and risk groups and the dates at which the coverage data were measured to construct a calendar as used in the epidemiological model.

#### Epidemiological model

The epidemiological model implemented in the package is an SEIR model, with two compartments for the Exposed and Infectious states that result in a more realistic gamma distributed average time for both the Exposed and Infectious states (rather than an exponentially distributed waiting time with single *E* and *I* compartments). The general model has the following form:

$$\begin{array}{c} \begin{array}{ccc} \frac{dS_{ik}}{dt} & = & -\lambda_{i}S_{ik}\\ \frac{dE_{ik}^{1}}{dt} & = & \lambda_{i}S_{ik}-\gamma_{1}E_{ik}^{1}\\ \frac{dE_{ik}^{2}}{dt} & = & \gamma_{1}\;(E_{ik}^{1}-E_{ik}^{2})\\ \frac{dI_{ik}^{1}}{dt} & = & \gamma_{1}E_{ik}^{2}-\gamma_{2}I_{ik}^{1}\\ \frac{dI_{ik}^{2}}{dt} & = & \gamma_{2}\;(I_{ik}^{1}-I_{ik}^{2})\\ \frac{dR_{ik}}{dt} & = & \gamma_{2}I_{ik}^{2} \end{array} \end{array}$$ (1)

where *S*_*ik*_ is the number of susceptibles in the age group *i* and risk group *k*, $E_{ik}^{1}$ and $E_{ik}^{2}$ are two compartments with exposed but not yet infectious individuals of age group *i* and risk group *k*, $I_{ik}^{1}$ and $I_{ik}^{2}$ represent infectious individuals, and immune individuals of age group *i* and risk group *k* are given by *R*_*ik*_. The overall rate of loss of latency and infectiousness are respectively given by *γ*_1_/2 and *γ*_2_/2, while the age-group-specific force of infection λ_*i*_ is given by

$$\begin{array}{c} \lambda_{i}=\sigma_{i}\;\sum_{j=1}^{x}\;\sum_{k=1}^{y}\;\beta_{i,j}\;(I_{jk}^{1}+I_{jk}^{2}) \end{array}$$ (2)

where *β*_*i*,*j*_ is the effective contact rate between individuals in age group *i* and age group *j*, and *σ*_*i*_ is the susceptibility of the age group *i* (that can be inferred from serological data) and *x* and *y* are the total number of age groups and risk groups, respectively. The effective contact rate is the transmission rate (Λ) multiplied with the probability of a contact between a individual in age group *i* and one in age group *j* (*C*_*i*,*j*_).

Eq 1 defines the trajectory of the infection for each age and risk group. To implement vaccination in the package we further separate each of the epidemiological compartments (SEEIIR) in the model (1) into vaccinated and non-vaccinated groups. Non-vaccinated people of age *i* and risk *k* are vaccinated at a given rate (*μ*_*ik*_) regardless of their epidemiological status. If the subject has already been exposed or infected, the infection progresses as normal. Depending on the efficacy of the vaccine (*α*_*i*_), a proportion of vaccinated susceptibles will become immune (*μ*_*ik*_*α*_*i*_*S*_*ik*_) and the total daily rate with which susceptible individuals become vaccinated and recovered is *μ*_*ik*_(*R*_*ik*_ + *α*_*i*_*S*_*ik*_). If the vaccine is not 100% effective a proportion of vaccinated individuals will become ‘vaccinated susceptibles’ (*μ*_*ik*_(1 − *α*_*i*_)*S*_*ik*_). For full details of the underlying model see the supplementary information of [6].

#### Inference

In addition to the numerically optimised epidemiological model we also implemented a highly optimised likelihood function. The basic likelihood function incorporated in the package has the following form for each age group (*i*):

$$L\left(n_{i}^{+},n_{i},m_{i}|\epsilon_{i},\psi ,\theta_{i}\right)=\sum_{m_{i}^{+}}\;L\left(n_{i}^{+},n_{i},m_{i}|m_{i}^{+},\theta_{i}\right)L\left(m_{i}^{+},m_{i}|\epsilon_{i},\psi ,\theta_{i}\right)$$

The likelihood of the data, given the ODE model, is separated into two parts. First, we assume that the virologically tested sera (*n*_*i*_) are a subsample of the people identified with an influenza-like-illness. As such, the number tested positive ($n_{i}^{+}$) is distributed as a hypergeometric function, with the number of people tested (*n*_*i*_), the number of people actually with that strain of influenza ($m_{i}^{+}$) and the total number of people (*m*_*i*_). The second part of the likelihood function defines how the values $m_{i}^{+}$ and *m*_*i*_ depend on the ascertainment probability (*ϵ*_*i*_), external inflow of influenza (*ψ*) and the epidemiological model parameters (*θ*_*i*_ = {Λ, *σ*_*i*_, *I*_0_}, with *I*_0_ the log transformed initial infected population: $I_{0}=\log I_{i}^{1}\left(0\right)$). Here, subscript *i* designates the age group *i* and the parameters (*ϵ*_*i*_; *ψ*; Λ; *σ*_*i*_; *I*_0_) are inferred from the data. We have no direct data for the number of people with a particular strain of influenza ($m_{i}^{+}$) so we integrate over all possibilities, resulting in a computationally intensive calculation. For full details of the likelihood and the method of optimisation see [6].

Finally, an advanced MCMC algorithm is used to generate samples from the posterior distribution of the parameter values using this likelihood function [[10]; Algorithm 6B].

The use of the hypergeometric distribution and the resulting computational complexity of the likelihood function above is due to the fact that the source of data on influenza incidence is dual, composed of the ILI data, which is an umbrella for several respiratory infections and virology testing which indicates which of these diseases is circulating. The actual incidence of one particular pathogen can thus only be inferred by combining these two sources. When direct data on disease prevalence are available the fluEvidenceSynthesis package can still be used, by replacing the above likelihood function with a simplified likelihood function. The vignettes of the package provide in-depth information on how to replace the likelihood function, with a user-specified function.

#### Forward modelling

The resulting posterior samples of the parameters can be used to model alternative interventions and explore the changes in the resulting outbreak size. This is generally done by adapting the previously fitted epidemiological model to account for the new intervention strategy. This model is then run using the posterior parameter samples and the final disease burden can be compared under the current scenario and/or under alternative scenarios. The disease burden can also be used to calculate the cost and benefits of the alternative scenarios.

#### Cost effectiveness

The package provides a number of functions to aid with calculating the cost effectiveness. Based on existing mortality rate and data on hospitalised cases, the incidence number is transformed into the number of consultations, hospitalisations and deaths using the function public_health_outcome(…). These numbers can then be converted into the costs and the benefits in reduced consultations, hospitalisations and mortality due to alternative intervention methods. It is also possible to calculate the number of vaccine doses needed using the vaccine_doses(…) function. Some of the rates and associated costs are country-specific as they depend on the healthcare system and therefore not included in the package.

## Results

We present an example examining the potential outbreak size reduction by extending the seasonal vaccination programme to include increased coverage in children aged 5-14 years. In this example we explore scenarios where either 40% or 80% coverage is achieved in the 5-14 year olds.

Following the workflow laid out in Fig 1 we can divide the needed process as follows:

1. Collect data for each season and subtype

The data available depends on the study area and collection agency, so this step is variable. Examples of the needed layout using UK data are provided with the package and can be used to base your data format on.

2. Run inference (for each season and subtype)

Performing the default parameter inference implemented in this package (see [6]) using user-specified data is straightforward by running the analysis using the inference(…) function, that needs to be run for each season and strain (as illustrated in the R (pseudo)code example below). Note that for performance reasons it is advised to adapt this pseudocode to run in parallel for each season and strain.

inference.results <- list()

for (season in seasons) {

 for (subtype in subtypes) {

  inference.results[[season]][[subtype]] <-

   inference(demography[[season]], ili[[season]][[subtype]], …)

 }

}

**Fig 2:**
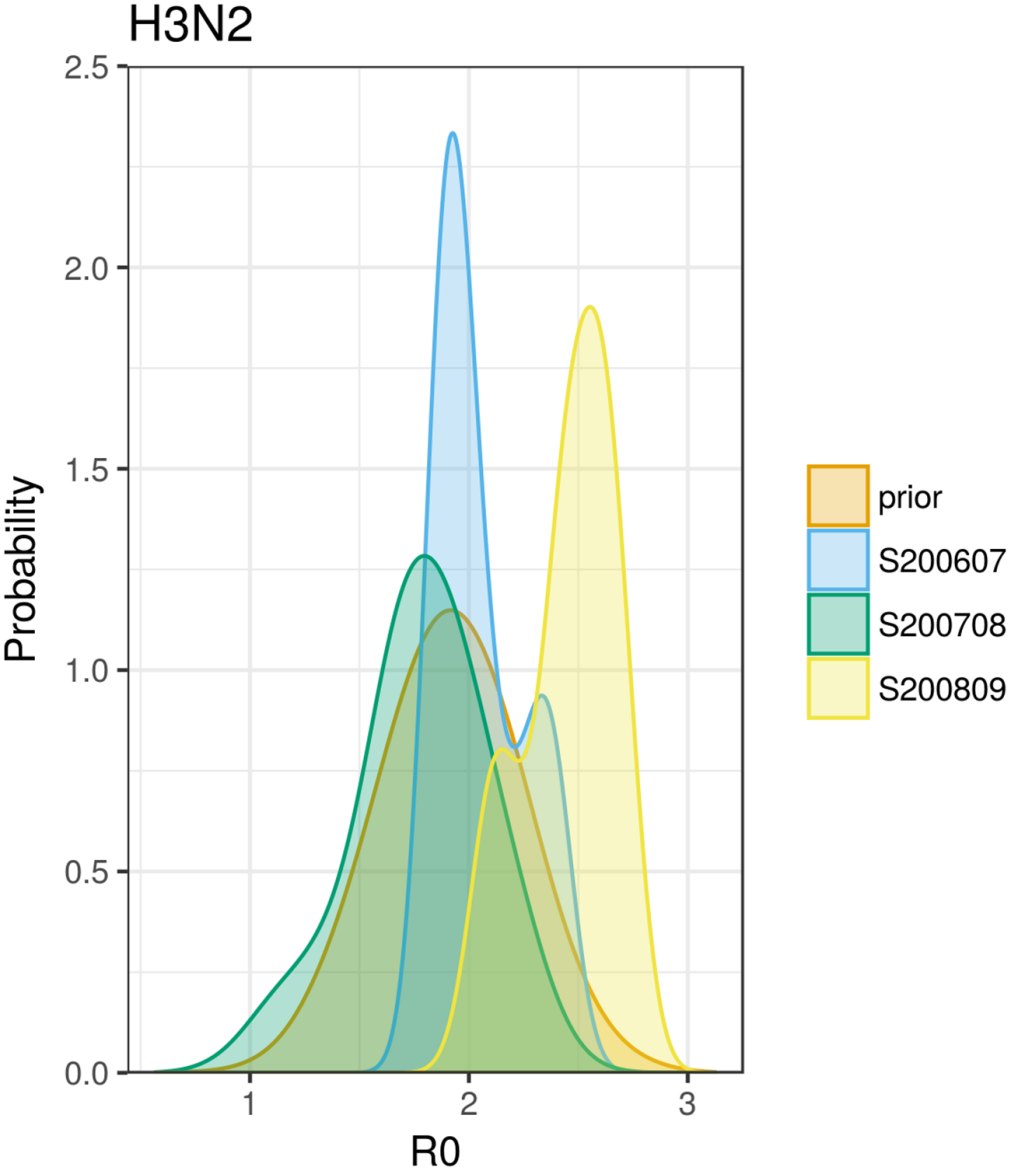
Posterior and prior probability of R0 for different seasons and serotype H3N2.

The inference(…) function is a high level function that implements the whole inference part of the workflow. This function takes all the data (as described earlier in this manuscript) and returns posterior samples of the parameters. The documentation of the package also shows how to adapt parts of the inference function for use with a different underlying epidemiological model, or different likelihood function. Fig 2 highlights the difference between our prior distribution of *R*_0_ and the posterior distribution of *R*_0_ following parameter inference. In the 2007/08 season the posterior is very similar to the prior. This is probably because in that season the incidence of H3N2 was very low compared to the other two seasons shown here.

3. Forward modelling

- Define alternative scenario

Any vaccination scenario depends on the efficacy of the vaccine, the total vaccine uptake, and the relative rate of uptake over time (e.g. during the first month the uptake rate in the UK is higher than in later months). To model our scenario we scaled the total uptake rate to 40% and 80%, while keeping the other factors constant. This was achieved by calculating the total uptake rate for the relevant age group (i.e. *V* = ∫ *v*(*t*)d*t*) and scaling up the original uptake rate to result in a total coverage of *V*_*s*_ = 0.4 or *V*_*s*_ = 0.8 (i.e. $\hat{v}\left(t\right)=V_{s}v\left(t\right)V$).

- Run vaccination scenario for all results with old vs. new scenario

**Fig 3:**
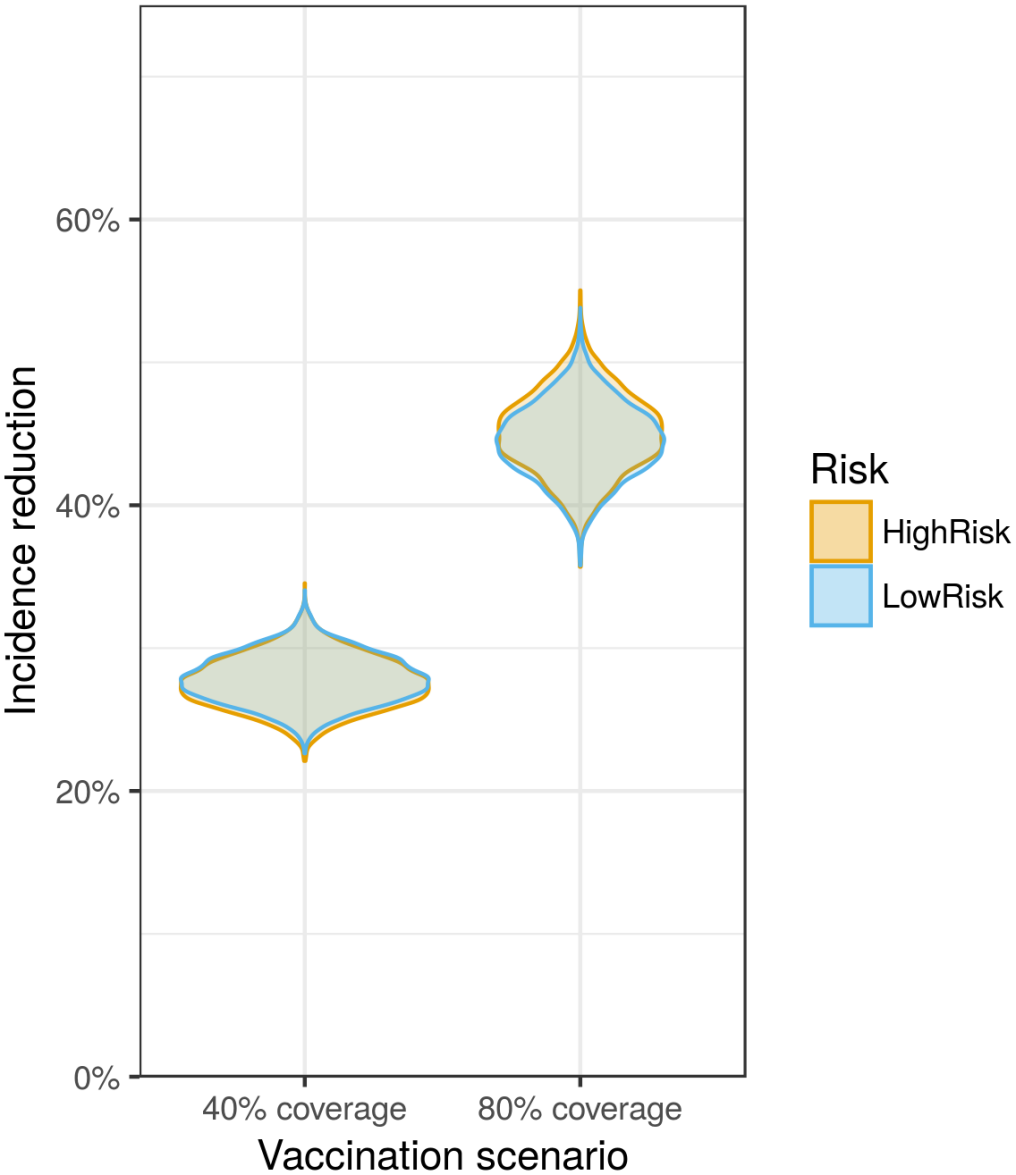
Reduction in influenza incidence under increased vaccination of the age group between 5 and 15. The orange colour signifies the reduction in patients classified as high risk. The blue colour is the reduction in the low risk population. The first results correspond to a scenario where 40 percent coverage is achieved, the second to a coverage of 80.

The inference step results in a number of posterior samples for the parameters and contact data. We can now use these samples and model the outcome of our alternative vaccination scenario. Then we calculate the difference in influenza burden between the original and our alternative vaccination scenarios. This is done by taking samples from each season and strain combination and calculating the disease burden over all the seasons/strains of the new scenario relative to the original scenario (using the vaccination_scenario(…) function). The results are summarized in Fig 3, showing a reduction of influenza cases in both the low and high risk groups under the new scenario. The magnitude of the reduction is dependent on the level of vaccine coverage. The higher coverage (80%) results in a reduction of around 5.9 million, while lower coverage (40%) results in a reduction of influenza cases of approximately 3.5 million (Fig 3).

4. Cost effectiveness

Performing the cost effectiveness analysis requires calculating the costs and benefits of the proposed intervention. The main cost in increasing vaccination stems from administering additional vaccine doses, which can be calculated with the vaccine_doses(…) function. Resulting benefits will mostly come from associated decreases in the number of consultations, reduced hospital admissions and finally reduced mortality. Generally, these values are calculated as the proportion of influenza cases that result in consultations, hospital admissions etc. The pseudocode below shows how to calculate the cost associated with a new vaccine calendar, assuming that 7% of infected people visit the GP and 0.2% and 2% of infected individuals are hospitalised for the low risk and high risk groups respectively.

posterior_cost <- rep(0, nbatch)

for (season in seasons) {

 #Vaccinepricecoversallsubtypes

 posterior_cost <- posterior_cost +

  rep(vaccine_price*

     (vaccine_doses(proposed_calender) - vaccine_doses(calendar)), nbatch)

 for (subtype in subtypes) {

  #Calculateincidenceofdifferentoutcomes

  outcomes <- rowSums(public_health_outcome(

   list("gp" = 0.07, "hospital" = c(0.002, 0.02)),

    vaccination_scenario(vaccine_calendar= calendar, …), …))

  #Calculateincidenceofdifferentoutcomesunderproposedpolicy

  new_outc <- rowSums(public_health_outcome(

   list("gp" = 0.07, "hospital" = c(0.002, 0.02)),

    vaccination_scenario(vaccine_calendar= proposed_calendar, …), …))

  #Differenceofcostundernewinterventionversusexistingpolicy

  posterior_cost <- posterior_cost +

   gp_cost*(new_outc$gp - outcomes$gp) +

   hospital_cost*(new_outc$hospital - outcomes$hospital)

 }

}

posterior_cost <- posterior_cost/length(seasons) #Perseason

**Fig 4:**
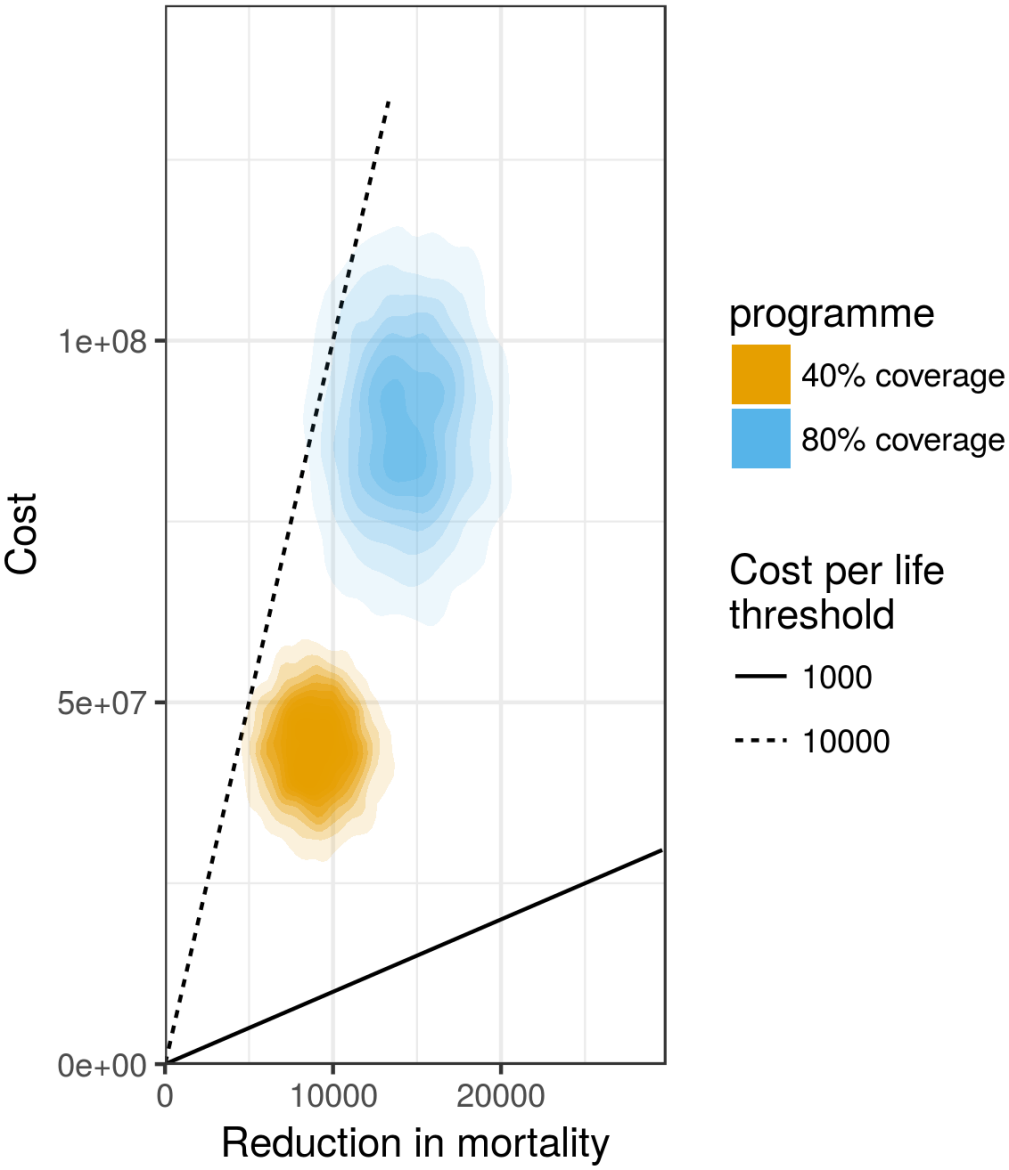
Cost effectiveness analysis showing the vaccine cost by reduction in mortality under two vaccination scenarios, taking into account uncertainty in vaccine cost, influenza incidence and death risk. The colours represent the uncertainty around the different scenarios. The lines represent two potential thresholds: (1) for a cost of up to 1000 for each life saved both programmes are not cost effective, (2) for a cost up to 10,000 per life saved both programmes are likely to be cost effective. The parameter values used in this figure (cost of vaccine: 17 ± 2.5 and mortality risk: 0.0025 ± 0.0005) were chosen for illustrative purposes only and should not be taken as realistic values.

Another important aspect of cost effectiveness calculations is the reduction of mortality due to incidence reduction. Fig 4 shows the cost of different vaccination scenarios as a function of reduction in mortality. More sophisticated cost effectiveness analysis could also take into account the risk and cost of hospitalisation as well as indirect effects due to increased vaccine-induced immunity in the population.

## Availability and future directions

The R package we presented here was developed to make advanced analysis of disease dynamics more approachable for data analysts and scientists interested in public health related questions. The package implemented the whole process of preparing the data, running the model, inferring posterior parameter distributions and performing cost effectiveness analysis. The approach implemented is based on the method by [6] and [7]. The package is modular in design, such that the method can be adapted by, for example, changing the underlying epidemiological model and/or likelihood function. While advanced methods such as implemented in this package are inherently complex and traditionally applied by specialised researchers, the package is designed to make the analysis accessible to a wider group of researchers. The package is freely available under the GPL-3.0 license on github at https://github.com/MJomaba/flu-evidence-synthesis.

### Contact matrix

Currently no explicit model of contact rates is included and inference of the contact matrix is performed by bootstrapping the available contact data [6]. One model we are working on is to use demographic data to inform contact inference, similar to the approach explored by [11]. Another simplification in the current approach is the assumption that the contact matrix is constant throughout the season. Indeed, contact matrices are dependent on behaviour and this can change during the year [12], either due to external drivers (e.g. school vacation) or in response to influenza (e.g. staying at home when sick [13]). These changes in behaviour could then be incorporated into the contact model. Due to the modularity of the package it is possible to plug-in such a contact model and explore its effect on intervention strategies.

### Multi-year

The package simulates each strain and season independently. An interesting possible addition would be to implement multi-year models, i.e. models where the current state is influenced by the previous years results. One possible approach would be to use the posterior of the previous season as a prior for the next season, but that would not capture dynamic effects of alternative vaccination strategies. Capturing long-term dynamic effects requires an explicit multi-year epidemiological model, which introduces challenges of modelling antigenic evolution of antigenic strains as well as waning of immunity [14–16]. Such a model would need to be calibrated jointly to multiple sources of data (e.g. DNA sequences, syndromic surveillance, serology, demography) [17].

### Beyond influenza

The package has been developed with a focus on influenza data, but could potentially be adapted to predict the impact and cost effectiveness for a range of interventions for other infectious diseases. The parts that are disease specific are the likelihood function and the transmission model. The transmission model is an SEIR model which can be used to capture a wide variety of diseases. If this model is not suitable for your analysis then it is of course possible to replace it with another model while still using the other parts of the package.

The likelihood function is suitable for any disease where most of the data on disease occurrence is a proxy for the actual occurrence, augmented with a smaller source of direct data (virological). If more direct data are available then it might be appropriate to replace the current likelihood function with a (simplified) likelihood that is only based on the direct data.

### Conclusion

The fluEvidenceSynthesis package implements the needed pipeline to go from raw epidemiological data to a cost effectiveness analysis based on Bayesian inference methods. The workflow implemented is based on the analysis used to predict the cost effectiveness of paediatric vaccination in the UK [6,7]. The package itself has been successfully used in further analyses, most notably [18] and [19]. Feedback from those analyses has been used to improve the package. It is implemented as a series of loosely connected steps and allows the researcher to replace any of these phases with their own method.
